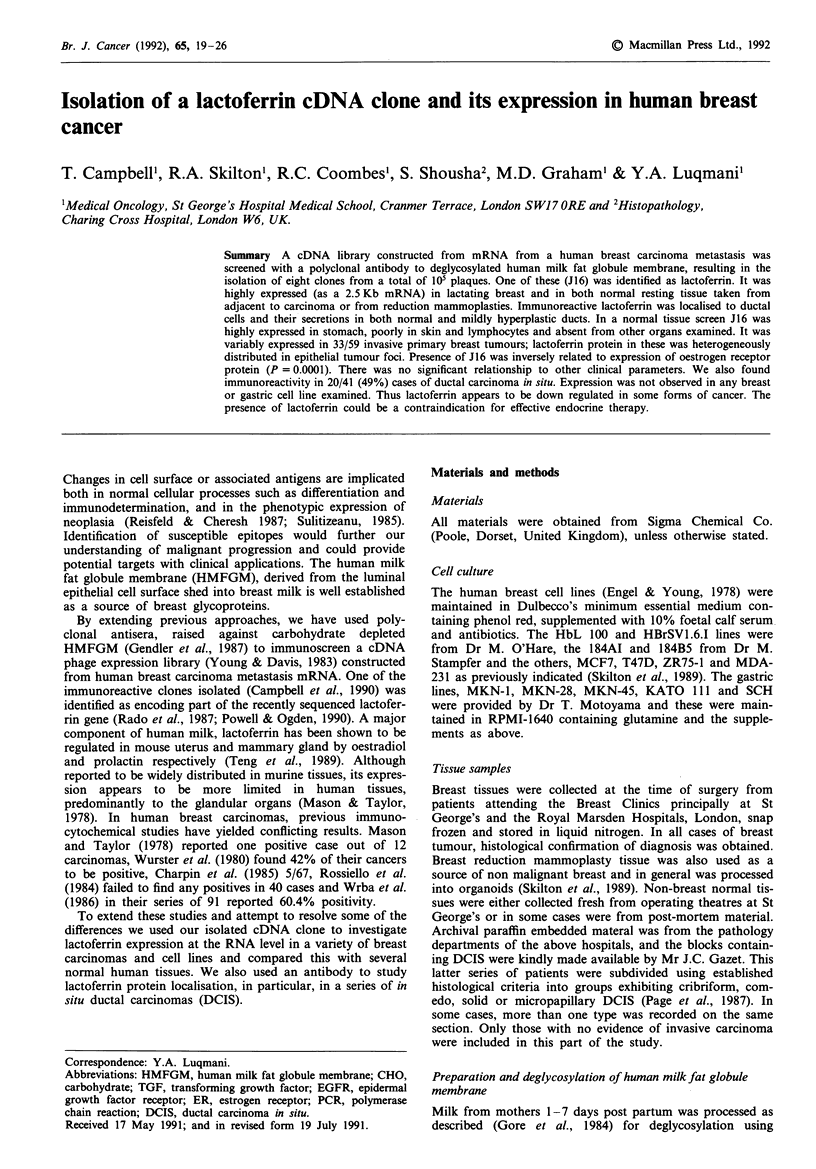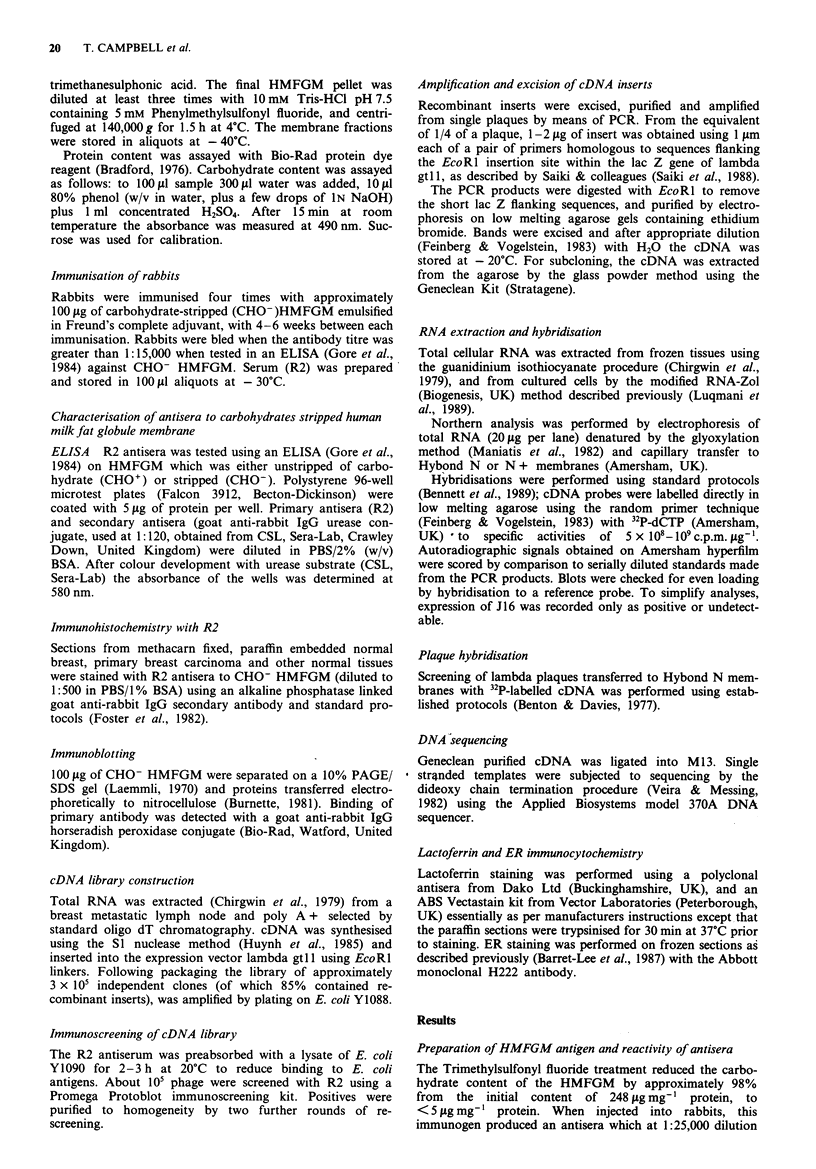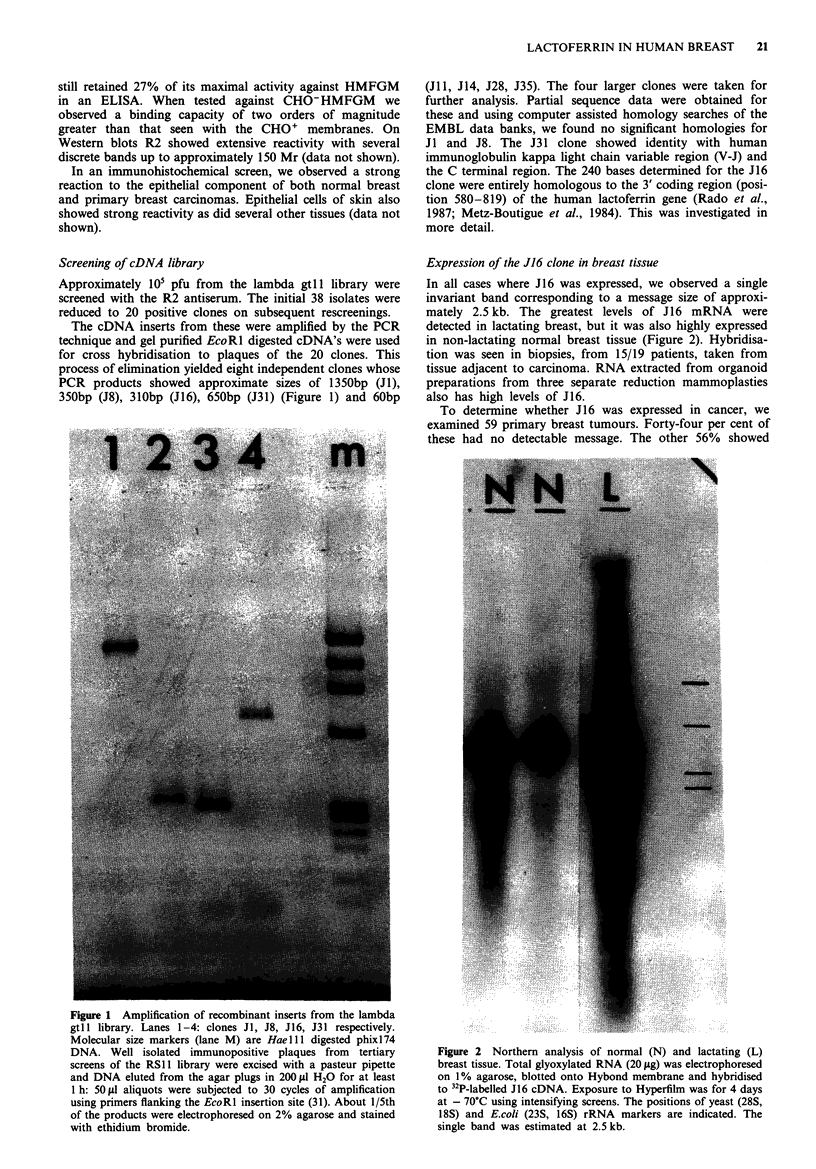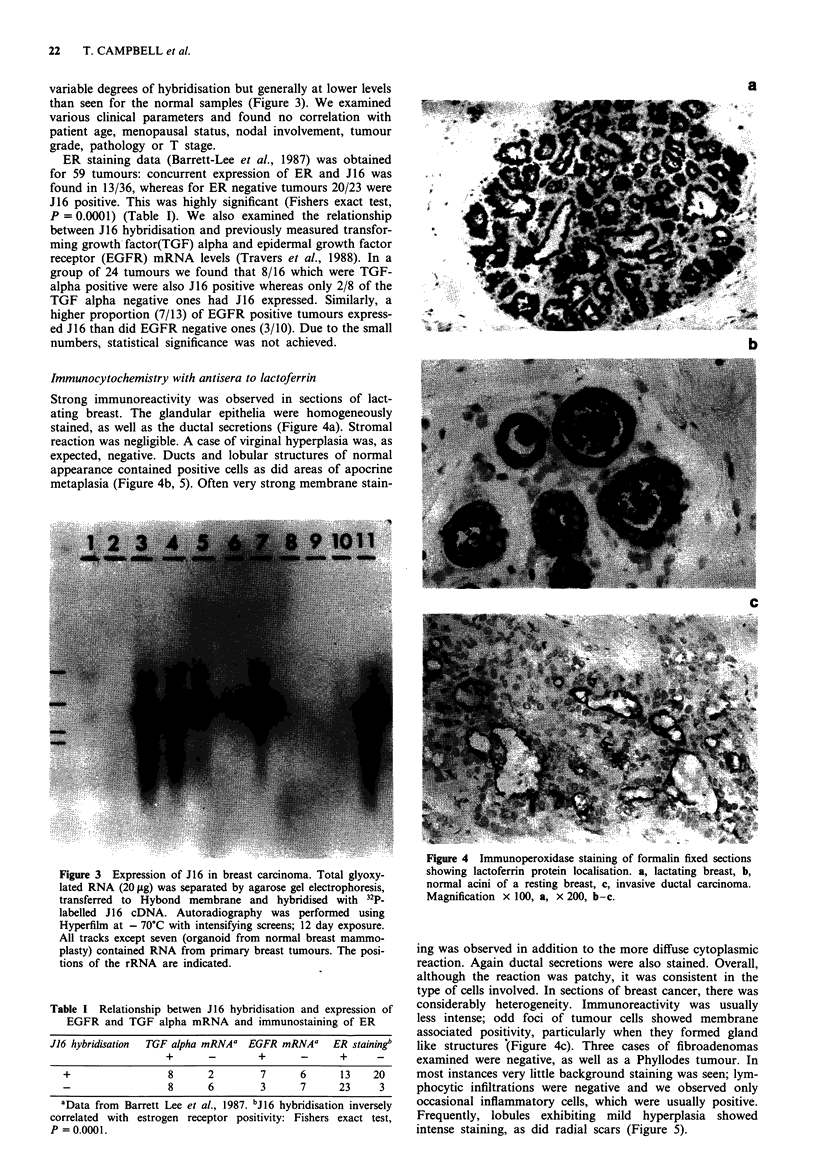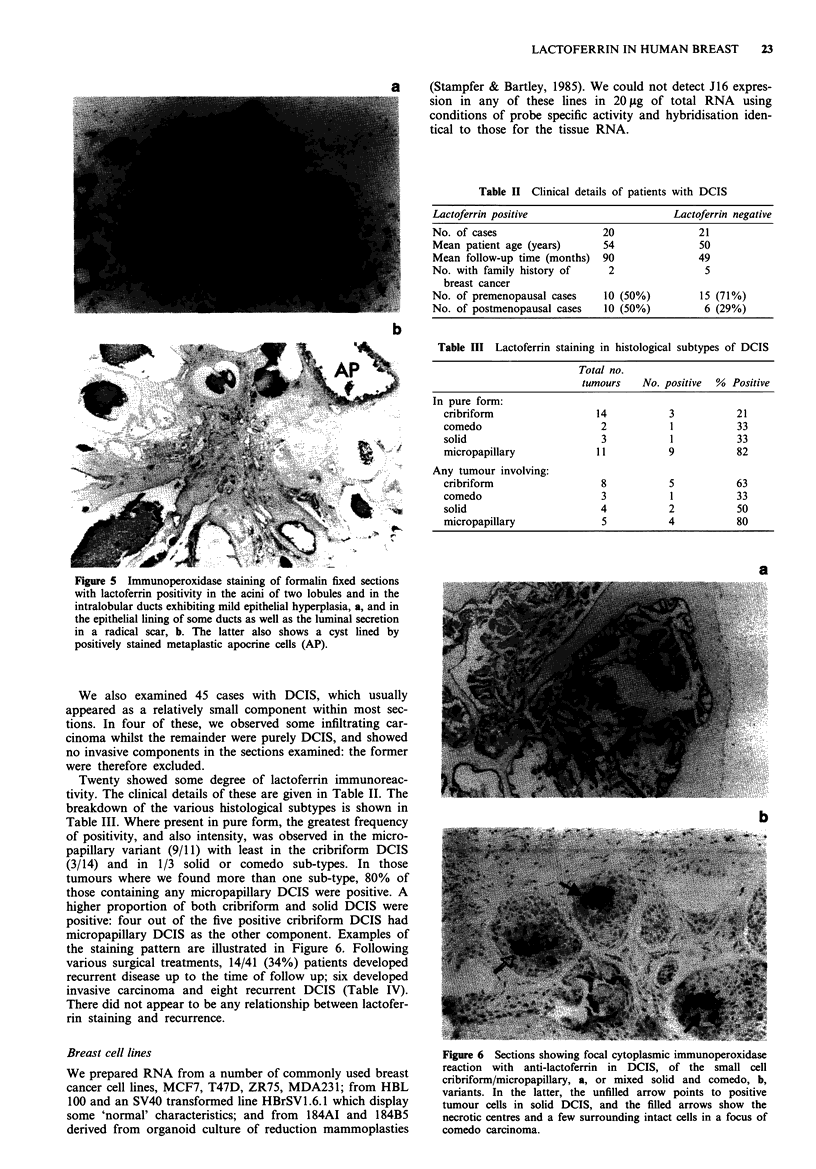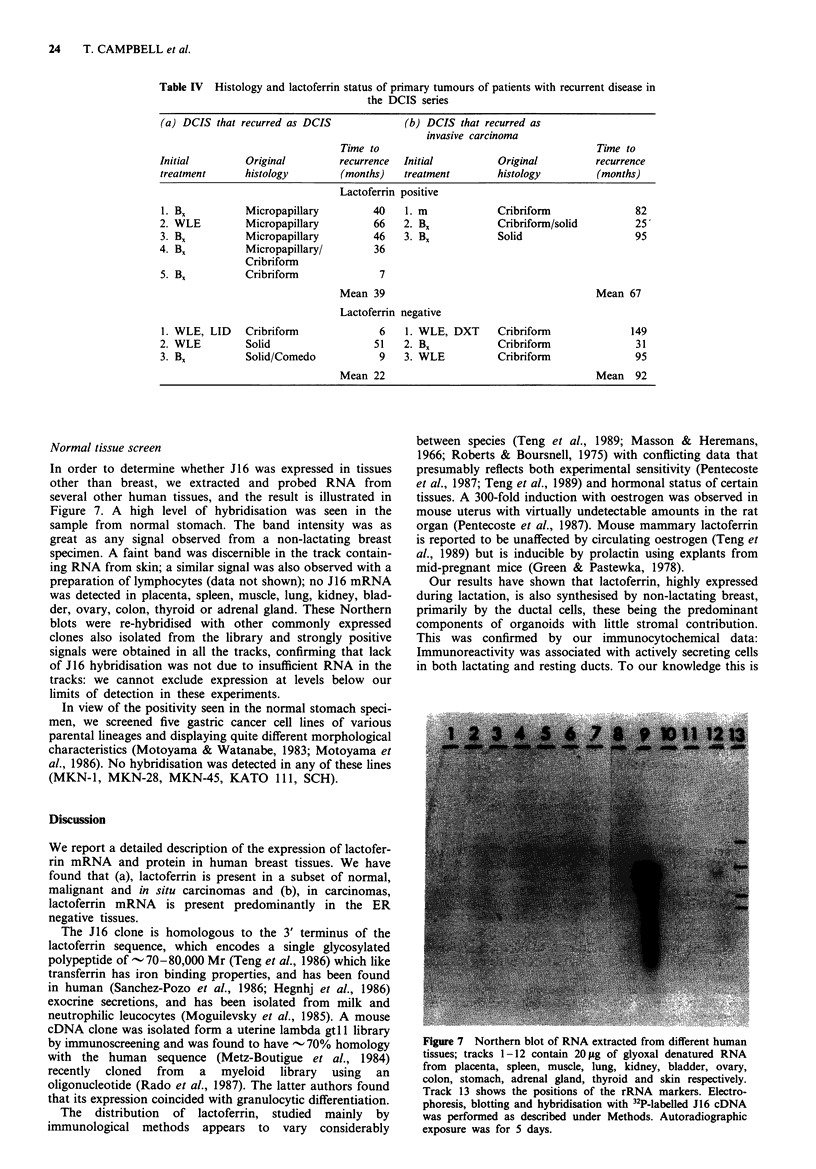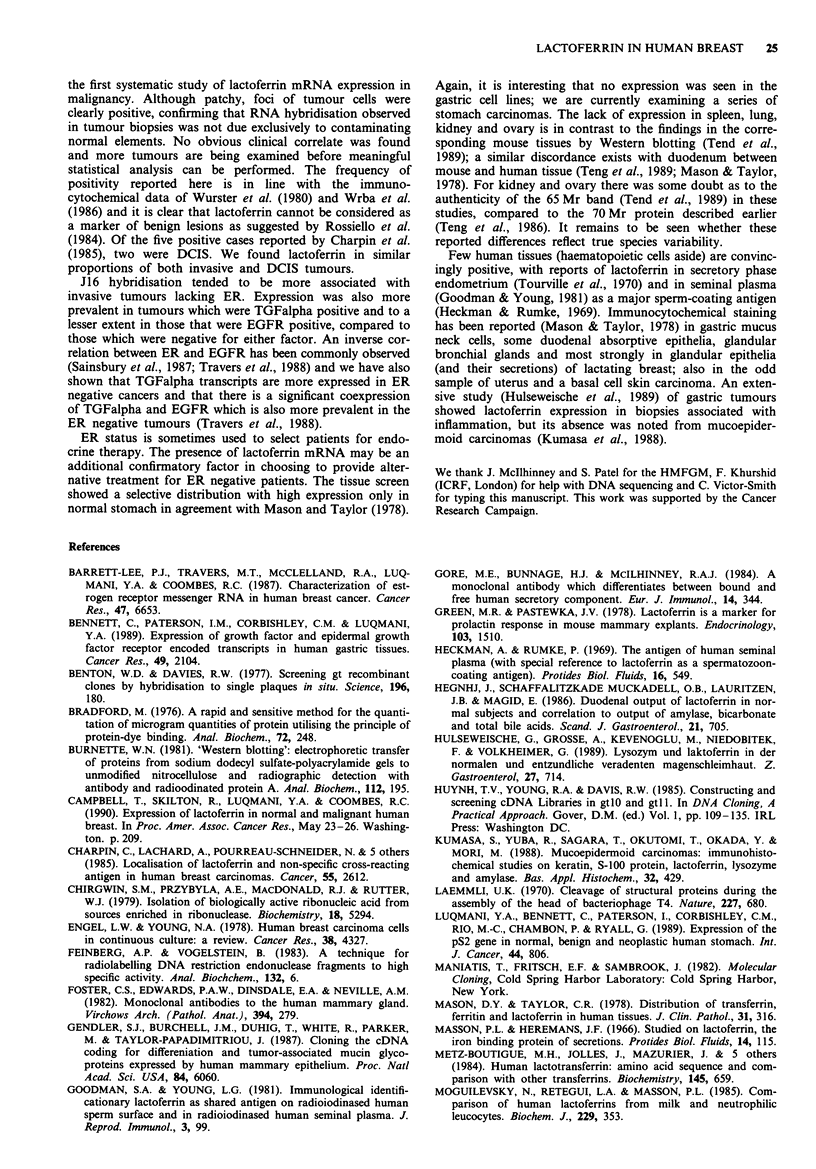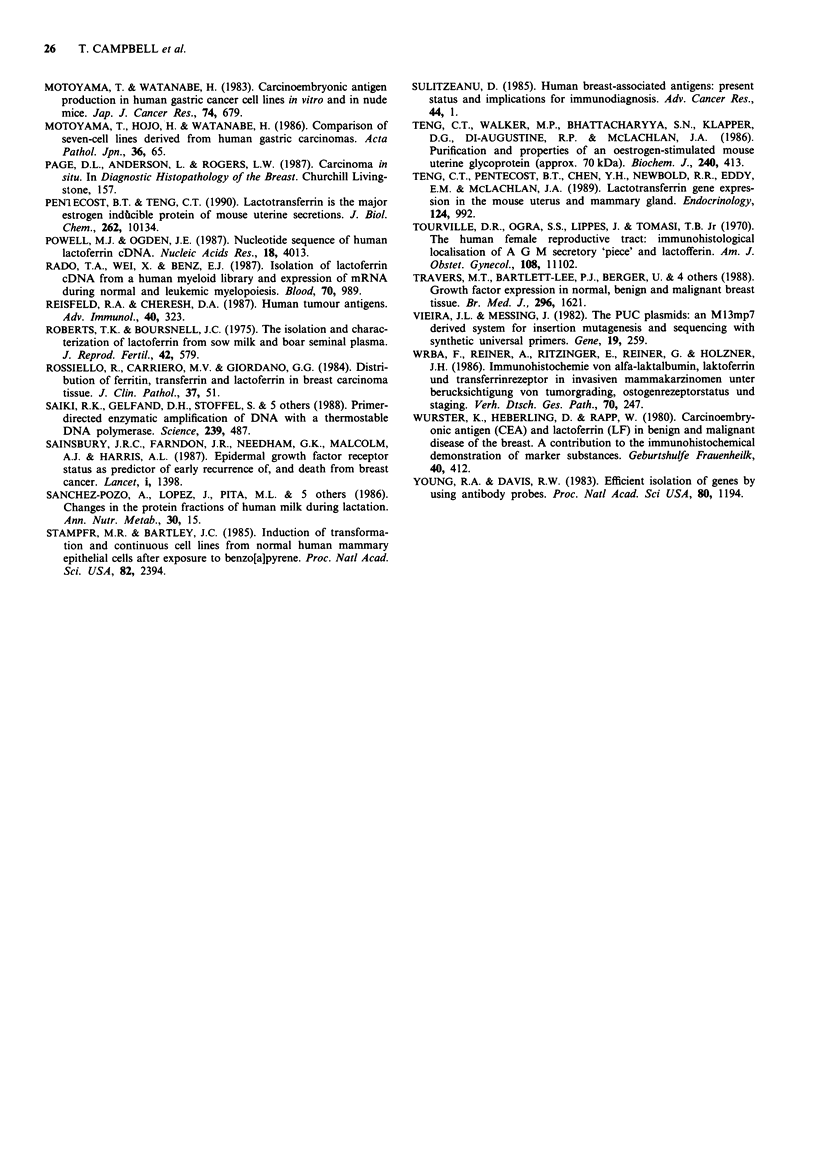# Isolation of a lactoferrin cDNA clone and its expression in human breast cancer.

**DOI:** 10.1038/bjc.1992.4

**Published:** 1992-01

**Authors:** T. Campbell, R. A. Skilton, R. C. Coombes, S. Shousha, M. D. Graham, Y. A. Luqmani

**Affiliations:** St George's Hospital Medical School, London, UK.

## Abstract

**Images:**


Br. J. Cancer (1992), 65, 19-26 © Macmillan Press Ltd., 1992

### Isolation of a lactoferrin cDNA clone and its expression in human breast cancer

T. Campbell[1], R.A. Skilton[1], R.C. Coombes[1], S. Shousha[2], M.D. Graham[1] & Y.A. Luqmani[1]

[1]*Medical Oncology, St George's Hospital Medical School, Cranmer Terrace, London SW17 0RE and* [2]*Histopathology, Charing Cross Hospital, London W6, UK.*

**Summary** A cDNA library constructed from mRNA from a human breast carcinoma metastasis was screened with a polyclonal antibody to deglycosylated human milk fat globule membrane, resulting in the isolation of eight clones from a total of $10^5$ plaques. One of these (J16) was identified as lactoferrin. It was highly expressed (as a 2.5 Kb mRNA) in lactating breast and in both normal resting tissue taken from adjacent to carcinoma or from reduction mammoplasties. Immunoreactive lactoferrin was localised to ductal cells and their secretions in both normal and mildly hyperplastic ducts. In a normal tissue screen J16 was highly expressed in stomach, poorly in skin and lymphocytes and absent from other organs examined. It was variably expressed in 33/59 invasive primary breast tumours; lactoferrin protein in these was heterogeneously distributed in epithelial tumour foci. Presence of J16 was inversely related to expression of oestrogen receptor protein ($P = 0.0001$). There was no significant relationship to other clinical parameters. We also found immunoreactivity in 20/41 (49%) cases of ductal carcinoma *in situ*. Expression was not observed in any breast or gastric cell line examined. Thus lactoferrin appears to be down regulated in some forms of cancer. The presence of lactoferrin could be a contraindication for effective endocrine therapy.

Changes in cell surface or associated antigens are implicated both in normal cellular processes such as differentiation and immunodetermination, and in the phenotypic expression of neoplasia (Reisfeld & Cheresh 1987; Sulitizeanu, 1985). Identification of susceptible epitopes would further our understanding of malignant progression and could provide potential targets with clinical applications. The human milk fat globule membrane (HMFGM), derived from the luminal epithelial cell surface shed into breast milk is well established as a source of breast glycoproteins.

By extending previous approaches, we have used polyclonal antisera, raised against carbohydrate depleted HMFGM (Gendler *et al.*, 1987) to immunoscreen a cDNA phage expression library (Young & Davis, 1983) constructed from human breast carcinoma metastasis mRNA. One of the immunoreactive clones isolated (Campbell *et al.*, 1990) was identified as encoding part of the recently sequenced lactoferrin gene (Rado *et al.*, 1987; Powell & Ogden, 1990). A major component of human milk, lactoferrin has been shown to be regulated in mouse uterus and mammary gland by oestradiol and prolactin respectively (Teng *et al.*, 1989). Although reported to be widely distributed in murine tissues, its expression appears to be more limited in human tissues, predominantly to the glandular organs (Mason & Taylor, 1978). In human breast carcinomas, previous immunocytochemical studies have yielded conflicting results. Mason and Taylor (1978) reported one positive case out of 12 carcinomas, Wurster *et al.* (1980) found 42% of their cancers to be positive, Charpin *et al.* (1985) 5/67, Rossiello *et al.* (1984) failed to find any positives in 40 cases and Wrba *et al.* (1986) in their series of 91 reported 60.4% positivity.

To extend these studies and attempt to resolve some of the differences we used our isolated cDNA clone to investigate lactoferrin expression at the RNA level in a variety of breast carcinomas and cell lines and compared this with several normal human tissues. We also used an antibody to study lactoferrin protein localisation, in particular, in a series of *in situ* ductal carcinomas (DCIS).

Correspondence: Y.A. Luqmani.
Abbreviations: HMFGM, human milk fat globule membrane; CHO, carbohydrate; TGF, transforming growth factor; EGFR, epidermal growth factor receptor; ER, estrogen receptor; PCR, polymerase chain reaction; DCIS, ductal carcinoma *in situ*.
Received 17 May 1991; and in revised form 19 July 1991.

### Materials and methods

### Materials

All materials were obtained from Sigma Chemical Co. (Poole, Dorset, United Kingdom), unless otherwise stated.

### Cell culture

The human breast cell lines (Engel & Young, 1978) were maintained in Dulbecco's minimum essential medium containing phenol red, supplemented with 10% foetal calf serum and antibiotics. The HbL 100 and HBrSV1.6.I lines were from Dr M. O'Hare, the 184AI and 184B5 from Dr M. Stampfer and the others, MCF7, T47D, ZR75-1 and MDA-231 as previously indicated (Skilton *et al.*, 1989). The gastric lines, MKN-1, MKN-28, MKN-45, KATO 111 and SCH were provided by Dr T. Motoyama and these were maintained in RPMI-1640 containing glutamine and the supplements as above.

### Tissue samples

Breast tissues were collected at the time of surgery from patients attending the Breast Clinics principally at St George's and the Royal Marsden Hospitals, London, snap frozen and stored in liquid nitrogen. In all cases of breast tumour, histological confirmation of diagnosis was obtained. Breast reduction mammoplasty tissue was also used as a source of non malignant breast and in general was processed into organoids (Skilton *et al.*, 1989). Non-breast normal tissues were either collected fresh from operating theatres at St George's or in some cases were from post-mortem material. Archival paraffin embedded materal was from the pathology departments of the above hospitals, and the blocks containing DCIS were kindly made available by Mr J.C. Gazet. This latter series of patients were subdivided using established histological criteria into groups exhibiting cribriform, comedo, solid or micropapillary DCIS (Page *et al.*, 1987). In some cases, more than one type was recorded on the same section. Only those with no evidence of invasive carcinoma were included in this part of the study.

### Preparation and deglycosylation of human milk fat globule membrane

Milk from mothers 1–7 days post partum was processed as described (Gore *et al.*, 1984) for deglycosylation using trimethanesulphonic acid. The final HMFGM pellet was diluted at least three times with 10 mM Tris-HCl pH 7.5 containing 5 mM Phenylmethylsulfonyl fluoride, and centrifuged at 140,000 *g* for 1.5 h at 4°C. The membrane fractions were stored in aliquots at − 40°C.

Protein content was assayed with Bio-Rad protein dye reagent (Bradford, 1976). Carbohydrate content was assayed as follows: to 100 µl sample 300 µl water was added, 10 µl 80% phenol (w/v in water, plus a few drops of 1N NaOH) plus 1 ml concentrated $H_2SO_4$. After 15 min at room temperature the absorbance was measured at 490 nm. Sucrose was used for calibration.

*Immunisation of rabbits*

Rabbits were immunised four times with approximately 100 µg of carbohydrate-stripped ($CHO^-$)HMFGM emulsified in Freund's complete adjuvant, with 4–6 weeks between each immunisation. Rabbits were bled when the antibody titre was greater than 1:15,000 when tested in an ELISA (Gore *et al.*, 1984) against $CHO^-$ HMFGM. Serum (R2) was prepared and stored in 100 µl aliquots at − 30°C.

*Characterisation of antisera to carbohydrates stripped human milk fat globule membrane*

*ELISA* R2 antisera was tested using an ELISA (Gore *et al.*, 1984) on HMFGM which was either unstripped of carbohydrate ($CHO^+$) or stripped ($CHO^-$). Polystyrene 96-well microtest plates (Falcon 3912, Becton-Dickinson) were coated with 5 µg of protein per well. Primary antisera (R2) and secondary antisera (goat anti-rabbit IgG urease conjugate, used at 1:120, obtained from CSL, Sera-Lab, Crawley Down, United Kingdom) were diluted in PBS/2% (w/v) BSA. After colour development with urease substrate (CSL, Sera-Lab) the absorbance of the wells was determined at 580 nm.

*Immunohistochemistry with R2*

Sections from methacarn fixed, paraffin embedded normal breast, primary breast carcinoma and other normal tissues were stained with R2 antisera to $CHO^-$ HMFGM (diluted to 1:500 in PBS/1% BSA) using an alkaline phosphatase linked goat anti-rabbit IgG secondary antibody and standard protocols (Foster *et al.*, 1982).

*Immunoblotting*

100 µg of $CHO^-$ HMFGM were separated on a 10% PAGE/SDS gel (Laemmli, 1970) and proteins transferred electrophoretically to nitrocellulose (Burnette, 1981). Binding of primary antibody was detected with a goat anti-rabbit IgG horseradish peroxidase conjugate (Bio-Rad, Watford, United Kingdom).

*cDNA library construction*

Total RNA was extracted (Chirgwin *et al.*, 1979) from a breast metastatic lymph node and poly A + selected by standard oligo dT chromatography. cDNA was synthesised using the S1 nuclease method (Huynh *et al.*, 1985) and inserted into the expression vector lambda gt11 using *Eco*R1 linkers. Following packaging the library of approximately $3 \times 10^5$ independent clones (of which 85% contained recombinant inserts), was amplified by plating on *E. coli* Y1088.

*Immunoscreening of cDNA library*

The R2 antiserum was preabsorbed with a lysate of *E. coli* Y1090 for 2–3 h at 20°C to reduce binding to *E. coli* antigens. About $10^5$ phage were screened with R2 using a Promega Protoblot immunoscreening kit. Positives were purified to homogeneity by two further rounds of rescreening.

*Amplification and excision of cDNA inserts*

Recombinant inserts were excised, purified and amplified from single plaques by means of PCR. From the equivalent of 1/4 of a plaque, 1–2 µg of insert was obtained using 1 µm each of a pair of primers homologous to sequences flanking the *Eco*R1 insertion site within the lac Z gene of lambda gt11, as described by Saiki & colleagues (Saiki *et al.*, 1988).

The PCR products were digested with *Eco*R1 to remove the short lac Z flanking sequences, and purified by electrophoresis on low melting agarose gels containing ethidium bromide. Bands were excised and after appropriate dilution (Feinberg & Vogelstein, 1983) with $H_2O$ the cDNA was stored at − 20°C. For subcloning, the cDNA was extracted from the agarose by the glass powder method using the Geneclean Kit (Stratagene).

*RNA extraction and hybridisation*

Total cellular RNA was extracted from frozen tissues using the guanidinium isothiocyanate procedure (Chirgwin *et al.*, 1979), and from cultured cells by the modified RNA-Zol (Biogenesis, UK) method described previously (Luqmani *et al.*, 1989).

Northern analysis was performed by electrophoresis of total RNA (20 µg per lane) denatured by the glyoxylation method (Maniatis *et al.*, 1982) and capillary transfer to Hybond N or N + membranes (Amersham, UK).

Hybridisations were performed using standard protocols (Bennett *et al.*, 1989); cDNA probes were labelled directly in low melting agarose using the random primer technique (Feinberg & Vogelstein, 1983) with $^{32}$P-dCTP (Amersham, UK) to specific activities of $5 \times 10^8 - 10^9$ c.p.m. $\mu g^{-1}$. Autoradiographic signals obtained on Amersham hyperfilm were scored by comparison to serially diluted standards made from the PCR products. Blots were checked for even loading by hybridisation to a reference probe. To simplify analyses, expression of J16 was recorded only as positive or undetectable.

*Plaque hybridisation*

Screening of lambda plaques transferred to Hybond N membranes with $^{32}$P-labelled cDNA was performed using established protocols (Benton & Davies, 1977).

*DNA sequencing*

Geneclean purified cDNA was ligated into M13. Single stranded templates were subjected to sequencing by the dideoxy chain termination procedure (Veira & Messing, 1982) using the Applied Biosystems model 370A DNA sequencer.

*Lactoferrin and ER immunocytochemistry*

Lactoferrin staining was performed using a polyclonal antisera from Dako Ltd (Buckinghamshire, UK), and an ABS Vectastain kit from Vector Laboratories (Peterborough, UK) essentially as per manufacturers instructions except that the paraffin sections were trypsinised for 30 min at 37°C prior to staining. ER staining was performed on frozen sections as described previously (Barret-Lee *et al.*, 1987) with the Abbott monoclonal H222 antibody.

### Results

*Preparation of HMFGM antigen and reactivity of antisera*

The Trimethylsulfonyl fluoride treatment reduced the carbohydrate content of the HMFGM by approximately 98% from the initial content of 248 µg $mg^{-1}$ protein, to < 5 µg $mg^{-1}$ protein. When injected into rabbits, this immunogen produced an antisera which at 1:25,000 dilution still retained 27% of its maximal activity against HMFGM in an ELISA. When tested against $CHO^-$HMFGM we observed a binding capacity of two orders of magnitude greater than that seen with the $CHO^+$ membranes. On Western blots R2 showed extensive reactivity with several discrete bands up to approximately 150 Mr (data not shown).

In an immunohistochemical screen, we observed a strong reaction to the epithelial component of both normal breast and primary breast carcinomas. Epithelial cells of skin also showed strong reactivity as did several other tissues (data not shown).

*Screening of cDNA library*

Approximately $10^5$ pfu from the lambda gt11 library were screened with the R2 antiserum. The initial 38 isolates were reduced to 20 positive clones on subsequent rescreenings.

The cDNA inserts from these were amplified by the PCR technique and gel purified *Eco*R1 digested cDNA's were used for cross hybridisation to plaques of the 20 clones. This process of elimination yielded eight independent clones whose PCR products showed approximate sizes of 1350bp (J1), 350bp (J8), 310bp (J16), 650bp (J31) (Figure 1) and 60bp (J11, J14, J28, J35). The four larger clones were taken for further analysis. Partial sequence data were obtained for these and using computer assisted homology searches of the EMBL data banks, we found no significant homologies for J1 and J8. The J31 clone showed identity with human immunoglobulin kappa light chain variable region (V-J) and the C terminal region. The 240 bases determined for the J16 clone were entirely homologous to the 3′ coding region (position 580–819) of the human lactoferrin gene (Rado *et al.*, 1987; Metz-Boutigue *et al.*, 1984). This was investigated in more detail.

**Figure 1** Amplification of recombinant inserts from the lambda gt11 library. Lanes 1–4: clones J1, J8, J16, J31 respectively. Molecular size markers (lane M) are *Hae*111 digested phix174 DNA. Well isolated immunopositive plaques from tertiary screens of the RS11 library were excised with a pasteur pipette and DNA eluted from the agar plugs in 200 µl $H_2O$ for at least 1 h: 50 µl aliquots were subjected to 30 cycles of amplification using primers flanking the *Eco*R1 insertion site (31). About 1/5th of the products were electrophoresed on 2% agarose and stained with ethidium bromide.

*Expression of the J16 clone in breast tissue*

In all cases where J16 was expressed, we observed a single invariant band corresponding to a message size of approximately 2.5 kb. The greatest levels of J16 mRNA were detected in lactating breast, but it was also highly expressed in non-lactating normal breast tissue (Figure 2). Hybridisation was seen in biopsies, from 15/19 patients, taken from tissue adjacent to carcinoma. RNA extracted from organoid preparations from three separate reduction mammoplasties also has high levels of J16.

To determine whether J16 was expressed in cancer, we examined 59 primary breast tumours. Forty-four per cent of these had no detectable message. The other 56% showed

**Figure 2** Northern analysis of normal (N) and lactating (L) breast tissue. Total glyoxylated RNA (20 µg) was electrophoresed on 1% agarose, blotted onto Hybond membrane and hybridised to $^{32}$P-labelled J16 cDNA. Exposure to Hyperfilm was for 4 days at − 70°C using intensifying screens. The positions of yeast (28S, 18S) and *E.coli* (23S, 16S) rRNA markers are indicated. The single band was estimated at 2.5 kb.

variable degrees of hybridisation but generally at lower levels than seen for the normal samples (Figure 3). We examined various clinical parameters and found no correlation with patient age, menopausal status, nodal involvement, tumour grade, pathology or T stage.

ER staining data (Barrett-Lee et al., 1987) was obtained for 59 tumours: concurrent expression of ER and J16 was found in 13/36, whereas for ER negative tumours 20/23 were J16 positive. This was highly significant (Fishers exact test, $P = 0.0001$) (Table I). We also examined the relationship between J16 hybridisation and previously measured transforming growth factor(TGF) alpha and epidermal growth factor receptor (EGFR) mRNA levels (Travers et al., 1988). In a group of 24 tumours we found that 8/16 which were TGF-alpha positive were also J16 positive whereas only 2/8 of the TGF alpha negative ones had J16 expressed. Similarly, a higher proportion (7/13) of EGFR positive tumours expressed J16 than did EGFR negative ones (3/10). Due to the small numbers, statistical significance was not achieved.

*Immunocytochemistry with antisera to lactoferrin*

Strong immunoreactivity was observed in sections of lactating breast. The glandular epithelia were homogeneously stained, as well as the ductal secretions (Figure 4a). Stromal reaction was negligible. A case of virginal hyperplasia was, as expected, negative. Ducts and lobular structures of normal appearance contained positive cells as did areas of apocrine metaplasia (Figure 4b, 5). Often very strong membrane staining was observed in addition to the more diffuse cytoplasmic reaction. Again ductal secretions were also stained. Overall, although the reaction was patchy, it was consistent in the type of cells involved. In sections of breast cancer, there was considerably heterogeneity. Immunoreactivity was usually less intense; odd foci of tumour cells showed membrane associated positivity, particularly when they formed gland like structures (Figure 4c). Three cases of fibroadenomas examined were negative, as well as a Phyllodes tumour. In most instances very little background staining was seen; lymphocytic infiltrations were negative and we observed only occasional inflammatory cells, which were usually positive. Frequently, lobules exhibiting mild hyperplasia showed intense staining, as did radial scars (Figure 5).

**Figure 3** Expression of J16 in breast carcinoma. Total glyoxylated RNA (20 μg) was separated by agarose gel electrophoresis, transferred to Hybond membrane and hybridised with $^{32}$P-labelled J16 cDNA. Autoradiography was performed using Hyperfilm at − 70°C with intensifying screens; 12 day exposure. All tracks except seven (organoid from normal breast mammoplasty) contained RNA from primary breast tumours. The positions of the rRNA are indicated.

**Figure 4** Immunoperoxidase staining of formalin fixed sections showing lactoferrin protein localisation. **a**, lactating breast, **b**, normal acini of a resting breast, **c**, invasive ductal carcinoma. Magnification × 100, **a**, × 200, **b**–**c**.

**Table I** Relationship betwen J16 hybridisation and expression of EGFR and TGF alpha mRNA and immunostaining of ER

| J16 hybridisation | TGF alpha mRNA[a] | | EGFR mRNA[a] | | ER staining[b] | |
|---|---|---|---|---|---|---|
| | + | − | + | − | + | − |
| + | 8 | 2 | 7 | 6 | 13 | 20 |
| − | 8 | 6 | 3 | 7 | 23 | 3 |

[a]Data from Barrett Lee et al., 1987. [b]J16 hybridisation inversely correlated with estrogen receptor positivity: Fishers exact test, $P = 0.0001$.

**Figure 5** Immunoperoxidase staining of formalin fixed sections with lactoferrin positivity in the acini of two lobules and in the intralobular ducts exhibiting mild epithelial hyperplasia, **a**, and in the epithelial lining of some ducts as well as the luminal secretion in a radical scar, **b**. The latter also shows a cyst lined by positively stained metaplastic apocrine cells (AP).

We also examined 45 cases with DCIS, which usually appeared as a relatively small component within most sections. In four of these, we observed some infiltrating carcinoma whilst the remainder were purely DCIS, and showed no invasive components in the sections examined: the former were therefore excluded.

Twenty showed some degree of lactoferrin immunoreactivity. The clinical details of these are given in Table II. The breakdown of the various histological subtypes is shown in Table III. Where present in pure form, the greatest frequency of positivity, and also intensity, was observed in the micropapillary variant (9/11) with least in the cribriform DCIS (3/14) and in 1/3 solid or comedo sub-types. In those tumours where we found more than one sub-type, 80% of those containing any micropapillary DCIS were positive. A higher proportion of both cribriform and solid DCIS were positive: four out of the five positive cribriform DCIS had micropapillary DCIS as the other component. Examples of the staining pattern are illustrated in Figure 6. Following various surgical treatments, 14/41 (34%) patients developed recurrent disease up to the time of follow up; six developed invasive carcinoma and eight recurrent DCIS (Table IV). There did not appear to be any relationship between lactoferrin staining and recurrence.

*Breast cell lines*

We prepared RNA from a number of commonly used breast cancer cell lines, MCF7, T47D, ZR75, MDA231; from HBL 100 and an SV40 transformed line HBrSV1.6.1 which display some 'normal' characteristics; and from 184AI and 184B5 derived from organoid culture of reduction mammoplasties (Stampfer & Bartley, 1985). We could not detect J16 expression in any of these lines in 20 μg of total RNA using conditions of probe specific activity and hybridisation identical to those for the tissue RNA.

**Table II** Clinical details of patients with DCIS

| | *Lactoferrin positive* | *Lactoferrin negative* |
|---|---|---|
| No. of cases | 20 | 21 |
| Mean patient age (years) | 54 | 50 |
| Mean follow-up time (months) | 90 | 49 |
| No. with family history of breast cancer | 2 | 5 |
| No. of premenopausal cases | 10 (50%) | 15 (71%) |
| No. of postmenopausal cases | 10 (50%) | 6 (29%) |

**Table III** Lactoferrin staining in histological subtypes of DCIS

| | *Total no. tumours* | *No. positive* | *% Positive* |
|---|---|---|---|
| In pure form: | | | |
| cribriform | 14 | 3 | 21 |
| comedo | 2 | 1 | 33 |
| solid | 3 | 1 | 33 |
| micropapillary | 11 | 9 | 82 |
| Any tumour involving: | | | |
| cribriform | 8 | 5 | 63 |
| comedo | 3 | 1 | 33 |
| solid | 4 | 2 | 50 |
| micropapillary | 5 | 4 | 80 |

**Figure 6** Sections showing focal cytoplasmic immunoperoxidase reaction with anti-lactoferrin in DCIS, of the small cell cribriform/micropapillary, **a**, or mixed solid and comedo, **b**, variants. In the latter, the unfilled arrow points to positive tumour cells in solid DCIS, and the filled arrows show the necrotic centres and a few surrounding intact cells in a focus of comedo carcinoma.

**Table IV** Histology and lactoferrin status of primary tumours of patients with recurrent disease in the DCIS series

| *(a) DCIS that recurred as DCIS* | | | *(b) DCIS that recurred as invasive carcinoma* | | |
|---|---|---|---|---|---|
| *Initial treatment* | *Original histology* | *Time to recurrence (months)* | *Initial treatment* | *Original histology* | *Time to recurrence (months)* |
| | | Lactoferrin | positive | | |
| 1. $B_x$ | Micropapillary | 40 | 1. m | Cribriform | 82 |
| 2. WLE | Micropapillary | 66 | 2. $B_x$ | Cribriform/solid | 25 |
| 3. $B_x$ | Micropapillary | 46 | 3. $B_x$ | Solid | 95 |
| 4. $B_x$ | Micropapillary/ Cribriform | 36 | | | |
| 5. $B_x$ | Cribriform | 7 | | | |
| | | Mean 39 | | | Mean 67 |
| | | Lactoferrin | negative | | |
| 1. WLE, LID | Cribriform | 6 | 1. WLE, DXT | Cribriform | 149 |
| 2. WLE | Solid | 51 | 2. $B_x$ | Cribriform | 31 |
| 3. $B_x$ | Solid/Comedo | 9 | 3. WLE | Cribriform | 95 |
| | | Mean 22 | | | Mean 92 |

*Normal tissue screen*

In order to determine whether J16 was expressed in tissues other than breast, we extracted and probed RNA from several other human tissues, and the result is illustrated in Figure 7. A high level of hybridisation was seen in the sample from normal stomach. The band intensity was as great as any signal observed from a non-lactating breast specimen. A faint band was discernible in the track containing RNA from skin; a similar signal was also observed with a preparation of lymphocytes (data not shown); no J16 mRNA was detected in placenta, spleen, muscle, lung, kidney, bladder, ovary, colon, thyroid or adrenal gland. These Northern blots were re-hybridised with other commonly expressed clones also isolated from the library and strongly positive signals were obtained in all the tracks, confirming that lack of J16 hybridisation was not due to insufficient RNA in the tracks: we cannot exclude expression at levels below our limits of detection in these experiments.

In view of the positivity seen in the normal stomach specimen, we screened five gastric cancer cell lines of various parental lineages and displaying quite different morphological characteristics (Motoyama & Watanabe, 1983; Motoyama *et al.*, 1986). No hybridisation was detected in any of these lines (MKN-1, MKN-28, MKN-45, KATO 111, SCH).

### Discussion

We report a detailed description of the expression of lactoferrin mRNA and protein in human breast tissues. We have found that (a), lactoferrin is present in a subset of normal, malignant and *in situ* carcinomas and (b), in carcinomas, lactoferrin mRNA is present predominantly in the ER negative tissues.

The J16 clone is homologous to the 3′ terminus of the lactoferrin sequence, which encodes a single glycosylated polypeptide of ~70–80,000 Mr (Teng *et al.*, 1986) which like transferrin has iron binding properties, and has been found in human (Sanchez-Pozo *et al.*, 1986; Hegnhj *et al.*, 1986) exocrine secretions, and has been isolated from milk and neutrophilic leucocytes (Moguilevsky *et al.*, 1985). A mouse cDNA clone was isolated form a uterine lambda gt11 library by immunoscreening and was found to have ~70% homology with the human sequence (Metz-Boutigue *et al.*, 1984) recently cloned from a myeloid library using an oligonucleotide (Rado *et al.*, 1987). The latter authors found that its expression coincided with granulocytic differentiation.

The distribution of lactoferrin, studied mainly by immunological methods appears to vary considerably between species (Teng *et al.*, 1989; Masson & Heremans, 1966; Roberts & Boursnell, 1975) with conflicting data that presumably reflects both experimental sensitivity (Pentecoste *et al.*, 1987; Teng *et al.*, 1989) and hormonal status of certain tissues. A 300-fold induction with oestrogen was observed in mouse uterus with virtually undetectable amounts in the rat organ (Pentecoste *et al.*, 1987). Mouse mammary lactoferrin is reported to be unaffected by circulating oestrogen (Teng *et al.*, 1989) but is inducible by prolactin using explants from mid-pregnant mice (Green & Pastewka, 1978).

Our results have shown that lactoferrin, highly expressed during lactation, is also synthesised by non-lactating breast, primarily by the ductal cells, these being the predominant components of organoids with little stromal contribution. This was confirmed by our immunocytochemical data: Immunoreactivity was associated with actively secreting cells in both lactating and resting ducts. To our knowledge this is

**Figure 7** Northern blot of RNA extracted from different human tissues; tracks 1–12 contain 20 μg of glyoxal denatured RNA from placenta, spleen, muscle, lung, kidney, bladder, ovary, colon, stomach, adrenal gland, thyroid and skin respectively. Track 13 shows the positions of the rRNA markers. Electrophoresis, blotting and hybridisation with $^{32}$P-labelled J16 cDNA was performed as described under Methods. Autoradiographic exposure was for 5 days.

the first systematic study of lactoferrin mRNA expression in malignancy. Although patchy, foci of tumour cells were clearly positive, confirming that RNA hybridisation observed in tumour biopsies was not due exclusively to contaminating normal elements. No obvious clinical correlate was found and more tumours are being examined before meaningful statistical analysis can be performed. The frequency of positivity reported here is in line with the immunocytochemical data of Wurster *et al.* (1980) and Wrba *et al.* (1986) and it is clear that lactoferrin cannot be considered as a marker of benign lesions as suggested by Rossiello *et al.* (1984). Of the five positive cases reported by Charpin *et al.* (1985), two were DCIS. We found lactoferrin in similar proportions of both invasive and DCIS tumours.

J16 hybridisation tended to be more associated with invasive tumours lacking ER. Expression was also more prevalent in tumours which were TGFalpha positive and to a lesser extent in those that were EGFR positive, compared to those which were negative for either factor. An inverse correlation between ER and EGFR has been commonly observed (Sainsbury *et al.*, 1987; Travers *et al.*, 1988) and we have also shown that TGFalpha transcripts are more expressed in ER negative cancers and that there is a significant coexpression of TGFalpha and EGFR which is also more prevalent in the ER negative tumours (Travers *et al.*, 1988).

ER status is sometimes used to select patients for endocrine therapy. The presence of lactoferrin mRNA may be an additional confirmatory factor in choosing to provide alternative treatment for ER negative patients. The tissue screen showed a selective distribution with high expression only in normal stomach in agreement with Mason and Taylor (1978). Again, it is interesting that no expression was seen in the gastric cell lines; we are currently examining a series of stomach carcinomas. The lack of expression in spleen, lung, kidney and ovary is in contrast to the findings in the corresponding mouse tissues by Western blotting (Tend *et al.*, 1989); a similar discordance exists with duodenum between mouse and human tissue (Teng *et al.*, 1989; Mason & Taylor, 1978). For kidney and ovary there was some doubt as to the authenticity of the 65 Mr band (Tend *et al.*, 1989) in these studies, compared to the 70 Mr protein described earlier (Teng *et al.*, 1986). It remains to be seen whether these reported differences reflect true species variability.

Few human tissues (haematopoietic cells aside) are convincingly positive, with reports of lactoferrin in secretory phase endometrium (Tourville *et al.*, 1970) and in seminal plasma (Goodman & Young, 1981) as a major sperm-coating antigen (Heckman & Rumke, 1969). Immunocytochemical staining has been reported (Mason & Taylor, 1978) in gastric mucus neck cells, some duodenal absorptive epithelia, glandular bronchial glands and most strongly in glandular epithelia (and their secretions) of lactating breast; also in the odd sample of uterus and a basal cell skin carcinoma. An extensive study (Hulseweische *et al.*, 1989) of gastric tumours showed lactoferrin expression in biopsies associated with inflammation, but its absence was noted from mucoepidermoid carcinomas (Kumasa *et al.*, 1988).

We thank J. McIlhinney and S. Patel for the HMFGM, F. Khurshid (ICRF, London) for help with DNA sequencing and C. Victor-Smith for typing this manuscript. This work was supported by the Cancer Research Campaign.

### References

BARRETT-LEE, P.J., TRAVERS, M.T., McCLELLAND, R.A., LUQMANI, Y.A. & COOMBES, R.C. (1987). Characterization of estrogen receptor messenger RNA in human breast cancer. *Cancer Res.*, **47**, 6653.

BENNETT, C., PATERSON, I.M., CORBISHLEY, C.M. & LUQMANI, Y.A. (1989). Expression of growth factor and epidermal growth factor receptor encoded transcripts in human gastric tissues. *Cancer Res.*, **49**, 2104.

BENTON, W.D. & DAVIES, R.W. (1977). Screening gt recombinant clones by hybridisation to single plaques *in situ*. *Science*, **196**, 180.

BRADFORD, M. (1976). A rapid and sensitive method for the quantitation of microgram quantities of protein utilising the principle of protein-dye binding. *Anal. Biochem.*, **72**, 248.

BURNETTE, W.N. (1981). 'Western blotting': electrophoretic transfer of proteins from sodium dodecyl sulfate-polyacrylamide gels to unmodified nitrocellulose and radiographic detection with antibody and radioodinated protein A. *Anal. Biochem.*, **112**, 195.

CAMPBELL, T., SKILTON, R., LUQMANI, Y.A. & COOMBES, R.C. (1990). Expression of lactoferrin in normal and malignant human breast. In *Proc. Amer. Assoc. Cancer Res.*, May 23–26. Washington. p. 209.

CHARPIN, C., LACHARD, A., POURREAU-SCHNEIDER, N. & 5 others (1985). Localisation of lactoferrin and non-specific cross-reacting antigen in human breast carcinomas. *Cancer*, **55**, 2612.

CHIRGWIN, S.M., PRZYBYLA, A.E., MACDONALD, R.J. & RUTTER, W.J. (1979). Isolation of biologically active ribonucleic acid from sources enriched in ribonuclease. *Biochemistry*, **18**, 5294.

ENGEL, L.W. & YOUNG, N.A. (1978). Human breast carcinoma cells in continuous culture: a review. *Cancer Res.*, **38**, 4327.

FEINBERG, A.P. & VOGELSTEIN, B. (1983). A technique for radiolabelling DNA restriction endonuclease fragments to high specific activity. *Anal. Biochchem.*, **132**, 6.

FOSTER, C.S., EDWARDS, P.A.W., DINSDALE, E.A. & NEVILLE, A.M. (1982). Monoclonal antibodies to the human mammary gland. *Virchows Arch. (Pathol. Anat.)*, **394**, 279.

GENDLER, S.J., BURCHELL, J.M., DUHIG, T., WHITE, R., PARKER, M. & TAYLOR-PAPADIMITRIOU, J. (1987). Cloning the cDNA coding for differeniation and tumor-associated mucin glycoproteins expressed by human mammary epithelium. *Proc. Natl Acad. Sci. USA*, **84**, 6060.

GOODMAN, S.A. & YOUNG, L.G. (1981). Immunological identificationary lactoferrin as shared antigen on radioiodinased human sperm surface and in radioiodinased human seminal plasma. *J. Reprod. Immunol.*, **3**, 99.

GORE, M.E., BUNNAGE, H.J. & McILHINNEY, R.A.J. (1984). A monoclonal antibody which differentiates between bound and free human secretory component. *Eur. J. Immunol.*, **14**, 344.

GREEN, M.R. & PASTEWKA, J.V. (1978). Lactoferrin is a marker for prolactin response in mouse mammary explants. *Endocrinology*, **103**, 1510.

HECKMAN, A. & RUMKE, P. (1969). The antigen of human seminal plasma (with special reference to lactoferrin as a spermatozooncoating antigen). *Protides Biol. Fluids*, **16**, 549.

HEGNHJ, J., SCHAFFALITZKADE MUCKADELL, O.B., LAURITZEN, J.B. & MAGID, E. (1986). Duodenal output of lactoferrin in normal subjects and correlation to output of amylase, bicarbonate and total bile acids. *Scand. J. Gastroenterol.*, **21**, 705.

HULSEWEISCHE, G., GROSSE, A., KEVENOGLU, M., NIEDOBITEK, F. & VOLKHEIMER, G. (1989). Lysozym und laktoferrin in der normalen und entzundliche veradenten magenschleimhaut. *Z. Gastroenterol*, **27**, 714.

HUYNH, T.V., YOUNG, R.A. & DAVIS, R.W. (1985). Constructing and screening cDNA Libraries in gt10 and gt11. In *DNA Cloning, A Practical Approach*. Gover, D.M. (ed.) Vol. 1, pp. 109–135. IRL Press: Washington DC.

KUMASA, S., YUBA, R., SAGARA, T., OKUTOMI, T., OKADA, Y. & MORI, M. (1988). Mucoepidermoid carcinomas: immunohistochemical studies on keratin, S-100 protein, lactoferrin, lysozyme and amylase. *Bas. Appl. Histochem.*, **32**, 429.

LAEMMLI, U.K. (1970). Cleavage of structural proteins during the assembly of the head of bacteriophage T4. *Nature*, **227**, 680.

LUQMANI, Y.A., BENNETT, C., PATERSON, I., CORBISHLEY, C.M., RIO, M.-C., CHAMBON, P. & RYALL, G. (1989). Expression of the pS2 gene in normal, benign and neoplastic human stomach. *Int. J. Cancer*, **44**, 806.

MANIATIS, T., FRITSCH, E.F. & SAMBROOK, J. (1982). *Molecular Cloning*, Cold Spring Harbor Laboratory: Cold Spring Harbor, New York.

MASON, D.Y. & TAYLOR, C.R. (1978). Distribution of transferrin, ferritin and lactoferrin in human tissues. *J. Clin. Pathol.*, **31**, 316.

MASSON, P.L. & HEREMANS, J.F. (1966). Studied on lactoferrin, the iron binding protein of secretions. *Protides Biol. Fluids*, **14**, 115.

METZ-BOUTIGUE, M.H., JOLLES, J., MAZURIER, J. & 5 others (1984). Human lactotransferrin: amino acid sequence and comparison with other transferrins. *Biochemistry*, **145**, 659.

MOGUILEVSKY, N., RETEGUI, L.A. & MASSON, P.L. (1985). Comparison of human lactoferrins from milk and neutrophilic leucocytes. *Biochem. J.*, **229**, 353.

MOTOYAMA, T. & WATANABE, H. (1983). Carcinoembryonic antigen production in human gastric cancer cell lines *in vitro* and in nude mice. *Jap. J. Cancer Res.*, **74**, 679.

MOTOYAMA, T., HOJO, H. & WATANABE, H. (1986). Comparison of seven-cell lines derived from human gastric carcinomas. *Acta Pathol. Jpn.*, **36**, 65.

PAGE, D.L., ANDERSON, L. & ROGERS, L.W. (1987). Carcinoma *in situ*. In *Diagnostic Histopathology of the Breast*. Churchill Livingstone, 157.

PENTECOST, B.T. & TENG, C.T. (1990). Lactotransferrin is the major estrogen inducible protein of mouse uterine secretions. *J. Biol. Chem.*, **262**, 10134.

POWELL, M.J. & OGDEN, J.E. (1987). Nucleotide sequence of human lactoferrin cDNA. *Nucleic Acids Res.*, **18**, 4013.

RADO, T.A., WEI, X. & BENZ, E.J. (1987). Isolation of lactoferrin cDNA from a human myeloid library and expression of mRNA during normal and leukemic myelopoiesis. *Blood*, **70**, 989.

REISFELD, R.A. & CHERESH, D.A. (1987). Human tumour antigens. *Adv. Immunol.*, **40**, 323.

ROBERTS, T.K. & BOURSNELL, J.C. (1975). The isolation and characterization of lactoferrin from sow milk and boar seminal plasma. *J. Reprod. Fertil.*, **42**, 579.

ROSSIELLO, R., CARRIERO, M.V. & GIORDANO, G.G. (1984). Distribution of ferritin, transferrin and lactoferrin in breast carcinoma tissue. *J. Clin. Pathol.*, **37**, 51.

SAIKI, R.K., GELFAND, D.H., STOFFEL, S. & 5 others (1988). Primer-directed enzymatic amplification of DNA with a thermostable DNA polymerase. *Science*, **239**, 487.

SAINSBURY, J.R.C., FARNDON, J.R., NEEDHAM, G.K., MALCOLM, A.J. & HARRIS, A.L. (1987). Epidermal growth factor receptor status as predictor of early recurrence of, and death from breast cancer. *Lancet*, **i**, 1398.

SANCHEZ-POZO, A., LOPEZ, J., PITA, M.L. & 5 others (1986). Changes in the protein fractions of human milk during lactation. *Ann. Nutr. Metab.*, **30**, 15.

STAMPFR, M.R. & BARTLEY, J.C. (1985). Induction of transformation and continuous cell lines from normal human mammary epithelial cells after exposure to benzo[a]pyrene. *Proc. Natl Acad. Sci. USA*, **82**, 2394.

SULITZEANU, D. (1985). Human breast-associated antigens: present status and implications for immunodiagnosis. *Adv. Cancer Res.*, **44**, 1.

TENG, C.T., WALKER, M.P., BHATTACHARYYA, S.N., KLAPPER, D.G., DI-AUGUSTINE, R.P. & MCLACHLAN, J.A. (1986). Purification and properties of an oestrogen-stimulated mouse uterine glycoprotein (approx. 70 kDa). *Biochem. J.*, **240**, 413.

TENG, C.T., PENTECOST, B.T., CHEN, Y.H., NEWBOLD, R.R., EDDY, E.M. & MCLACHLAN, J.A. (1989). Lactotransferrin gene expression in the mouse uterus and mammary gland. *Endocrinology*, **124**, 992.

TOURVILLE, D.R., OGRA, S.S., LIPPES, J. & TOMASI, T.B. Jr (1970). The human female reproductive tract: immunohistological localisation of A G M secretory 'piece' and lactofferin. *Am. J. Obstet. Gynecol.*, **108**, 11102.

TRAVERS, M.T., BARTLETT-LEE, P.J., BERGER, U. & 4 others (1988). Growth factor expression in normal, benign and malignant breast tissue. *Br. Med. J.*, **296**, 1621.

VIEIRA, J.L. & MESSING, J. (1982). The PUC plasmids: an M13mp7 derived system for insertion mutagenesis and sequencing with synthetic universal primers. *Gene*, **19**, 259.

WRBA, F., REINER, A., RITZINGER, E., REINER, G. & HOLZNER, J.H. (1986). Immunohistochemie von alfa-laktalbumin, laktoferrin und transferrinrezeptor in invasiven mammakarzinomen unter berucksichtigung von tumorgrading, ostogenrezeptorstatus und staging. *Verh. Dtsch. Ges. Path.*, **70**, 247.

WURSTER, K., HEBERLING, D. & RAPP, W. (1980). Carcinoembryonic antigen (CEA) and lactoferrin (LF) in benign and malignant disease of the breast. A contribution to the immunohistochemical demonstration of marker substances. *Geburtshulfe Frauenheilk*, **40**, 412.

YOUNG, R.A. & DAVIS, R.W. (1983). Efficient isolation of genes by using antibody probes. *Proc. Natl Acad. Sci USA*, **80**, 1194.